# Does patient size matter when comparing the operation time between single-incision and conventional laparoscopic sleeve gastrectomy?

**DOI:** 10.1097/JS9.0000000000001168

**Published:** 2024-02-12

**Authors:** Cheuk-Kwan Sun, I-Wen Chen, I-Ting Tsai, Kuo-Chuan Hung

**Affiliations:** a Department of Emergency Medicine, E-Da Dachang Hospital, I-Shou University; b School of Medicine, College of Medicine, I-Shou University; c Department of Anesthesiology, Chi Mei Medical Center, Liouying, Tainan City; d Department of Emergency Medicine, E-Da Hospital, I-Shou University, Kaohsiung city, Taiwan

*Dear Editor*,

We read with great interest the article by Jiang *et al*.^1^ titled ‘Trocar number and placement for laparoscopic sleeve gastrectomy and comparison of single-incision and conventional laparoscopic sleeve gastrectomy: a systematic review and meta-analysis’ published in the *International Journal of Surgery*. The authors performed a systematic review and meta-analysis comparing single-incision laparoscopic sleeve gastrectomy (SLSG) with conventional laparoscopic sleeve gastrectomy (CLSG) for the treatment of morbid obesity^1^. Their review included 61 studies with over 20 000 patients. For CLSG procedures, they found that most studies utilized either four or five trocars arranged in an inverted trapezoid pattern, favoring the left side. A comparison between SLSG and CLSG showed a significantly longer operative time for SLSG but with lower pain scores on postoperative day 1^1^. There were no differences in other perioperative outcomes such as complications^1^. This is an important area of research, as bariatric surgery is gaining popularity as an effective treatment for obesity-related diseases. Understanding the optimal techniques, including trocar placement patterns and comparisons of different laparoscopic approaches, will allow for safer and more effective surgery. As SLSG is still an emerging technique, evaluating its risks and benefits compared with the current standard CLSG is particularly valuable. The authors^1^ should be congratulated on a robust systematic review and meta-analysis that contributes important new data to the literature.

However, the finding^1^ that operative time was significantly longer in SLSG than in CLSG implies that SLSG is likely a more technically challenging procedure that requires a longer learning curve for surgeons and may not yet be ready to fully replace CLSG. This suggests the need for additional surgical training and expertise in SLSG before it can be widely recommended. Furthermore, this finding emphasizes the need for careful selection of patients. Given the variations in body mass index (BMI) across the studies (i.e. 33.5–53.8 kg/m^2^ in patients undergoing SLSG) included in the original meta-analysis^1^, we hypothesized that BMI could be a factor contributing to the high heterogeneity (i.e. *I* ^2^=92%) in surgical times. Indeed, a previous large-scale clinical study involving 434 patients undergoing laparoscopic bariatric surgery has shown a significant positive association between BMI and operation time^2^. To investigate this hypothesis, we conducted a meta-regression analysis to determine if BMI is a covariate of surgical time based on the raw data from the original meta-analysis^1^ processed with the Comprehensive Meta-Analysis software. As depicted in Figure 1, we found no significant relationship between BMI and surgical time (coefficient: 0.51; *P*=0.46). Therefore, other factors, such as the surgeon’s experience with bariatric procedures, may be the underlying cause of the considerable heterogeneity observed.

**Figure 1:**
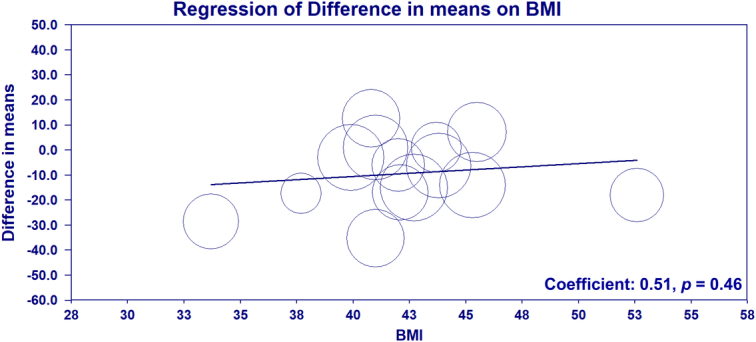
Meta-regression scatter plot illustrating the relationship between body mass index (BMI) and the difference in surgical time. Each circle represents a study included in the meta-analysis, with the size of the circle proportional to the weight of the study. The regression line indicates no significant association between BMI and surgical time (coefficient: 0.51, *P*=0.46), suggesting that other factors may contribute to the variability (*I* ^2^=92%) in surgical durations observed across different studies.

In summary, albeit promising, SLSG requires further evaluation, training of surgeons, and technique/technology advancement before it can be considered an equivalent or preferable alternative to the current standard CLSG procedure. Longer operative times imply that there is still a significant learning curve.

## Ethical approval

Not applicable.

## Consent

Not applicable.

## Sources of funding

No external funding was received for this study.

## Author contribution

K.-C.H. and C.-K.S.: wrote the main manuscript text; I-T.T. and I-W.C.: prepared Figure 1. All authors read and approved the final version of the manuscript.

## Conflicts of interest disclosure

The authors declare no conflicts of interest.

## Research registration unique identifying number (UIN)

Not applicable.

## Guarantor

Kuo-Chuan Hung.

## Data availability statement

The datasets used and/or analyzed in the current study are available from the corresponding author upon reasonable request.

## Provenance and peer review

This paper was not invited.
